# miR-196b-5p and miR-107 Expression Differentiates Ocular Sebaceous Carcinoma from Squamous Cell Carcinoma of the Conjunctiva

**DOI:** 10.3390/ijms23094877

**Published:** 2022-04-28

**Authors:** Ronald O. B. de Keizer, Anne L. M. Vriends, Gijsbert J. Hötte, Dion A. Paridaens, Erik A. C. Wiemer, Robert M. Verdijk

**Affiliations:** 1 Rotterdam Eye Hospital, Schiedamse Vest 180, 3011 BH Rotterdam, The Netherlands; r.dekeizer@oogziekenhuis.nl (R.O.B.d.K.); g.hotte@oogziekenhuis.nl (G.J.H.); d.paridaens@oogziekenhuis.nl (D.A.P.); 2 Department of Ophthalmology, Erasmus MC University Medical Center Rotterdam, Doctor Molewaterplein 40, 3015 GD Rotterdam, The Netherlands; 3 Department of Medical Oncology, Erasmus MC Cancer Institute, University Medical Center Rotterdam, Doctor Molewaterplein 40, 3015 GD Rotterdam, The Netherlands; a.vriends@erasmusmc.nl (A.L.M.V.); e.wiemer@erasmusmc.nl (E.A.C.W.); 4 Section Ophthalmic Pathology, Department of Pathology, Erasmus MC University Medical Center Rotterdam, Doctor Molewaterplein 40, 3015 GD Rotterdam, The Netherlands

**Keywords:** conjunctiva, eyelid, Ocular Surface Squamous Neoplasm(OSSN), Squamous Cell Carcinoma of the Conjunctiva (SCCC), Ocular Sebaceous Carcinoma (OSC), microRNA

## Abstract

An Ocular Sebaceous Carcinoma (OSC) is a rare malignant tumor for which initial clinical and pathological diagnosis is often incorrect. OSCs can mimic Squamous Cell Carcinomas of the Conjunctiva (SCCC). The aim of this study was to find microRNA biomarkers to distinguish OSCs and SCCCs from normal tissue and from each other. Clinical OSC and SCCC case files and the corresponding histopathological slides were collected and reviewed. Micro dissected formalin-fixed paraffin-embedded tumor and control tissues were subjected to semi-high throughput microRNA profiling. MicroRNA expression distinguishes OSCs and SCCCs from corresponding control tissues. Selected differentially expressed miRNAs were validated using single RT-PCR assays. No prognostic miRNAs could be identified that reliably predict SCCC metastasis or OSC recurrence. A comparison between OSCs (*n* = 14) and SCCCs (*n* = 18) revealed 38 differentially expressed microRNAs (*p* < 0.05). Differentially expressed miRNAs were selected for validation in the discovery cohort and an independent validation cohort (OSCs, *n* = 11; SCCCs, *n* = 12). At least two miRNAs, miR-196b-5p (*p* ≤ 0.05) and miR-107 (*p* ≤ 0.001), displayed a statistically significant differential expression between OSCs and SCCCs with miR-196b-5p upregulated in SCCCs and miR-107 upregulated in OSCs. In the validation cohort, microRNA miR-493-3p also showed significant upregulation in SCCCs when compared to OSCs (*p* ≤ 0.05). ROC analyses indicated that the combined miR-196b-5p and miR-107 expression levels predicted OSCs with 90.0% sensitivity and 83.3% specificity. In conclusion, the combined testing of miR-196b-5p and miR-107, can be of additional use in routine diagnostics to discriminate OSCs from SCCCs.

## 1. Introduction

Squamous Cell Carcinoma of the Conjunctiva (SCCC) is the most common epithelial neoplasm of the conjunctiva, with an incidence between 0.03 and 0.84 per 100,000 persons per year [1,2] in high-latitude areas with an increasing incidence rate estimated up to 1.8 per 100,000 persons per year in countries closer to the equator [3]. SCCCs are part of the spectrum of Ocular Surface Squamous Neoplasms (OSSN) and generally derive from Conjunctival Intraepithelial Neoplasia’s (CIN) or periocular Bowens disease [4]. Tumor recurrence has been demonstrated in 12–20% of cases [5,6], metastasis in up to 13%, and mortality in 5% [7,8].

An ocular sebaceous carcinoma (OSC) is a malignant tumor originating from sebaceous glands in the eyelid, caruncle, and conjunctiva [4]. The incidence is estimated between 0.5 and 0.8 per million persons per year for the periocular region [9,10]. OSCs may have aspecific clinical features and can mimic chalazia, blepharitis, conjunctivitis, and SCCC. As such, OSCs are known as the “great masqueraders”, causing delays of 1–3 years before a correct diagnosis is established [11]. A common characteristic of OSC is pagetoid spread, involving intraepithelial extension into the eyelid epidermis and conjunctival epithelium mimicking CIN and Bowens disease [11]. OSCs are relatively aggressive, and tumor recurrence has been demonstrated in 18% of cases, metastasis in 7–21%, and mortality in 6–20% [12,13].

OSC is a rare disease in which the initial pathologic diagnosis is often incorrect. Misdiagnosis has been reported in 40–75 percent of cases, depending on the experience of the pathologist and the suspicion of the referring surgeon [11]. In our clinic, residents in ophthalmology are taught to take a biopsy when performing an incision in a recurrent chalazion or chalazion without lipogranulomatous production. These biopsies (of the tarsal plate and palpebral conjunctiva) need to be sent into a pathologist fresh, for Oil-red-O staining (which can only be performed on fresh tissue as formalin dissolves the sebaceous material) [11,14]. Immunohistochemical staining’s for, e.g., androgen receptors, EMA, BerEp4, and adipophilin, have been reported to aid in differentiating sebaceous carcinomas from other neoplasms. However, these stains are known to provide difficulties in the distinction of less differentiated OSCs from SCCCs [14,15].

Both SCCC and OSC show overlap and differences in clinical behaviour and in their molecular genetic profiles. Recurrent *TP53*, *CDKN2A*, *RB1*, and *TERT* promoter mutations occur in both tumor types [16,17,18,19]. For both tumor types, HPV infection is responsible for about 10% of cases in the Western population [16,20]. We hypothesize that microRNA (miRNA) expression profiling may aid in establishing a differential diagnosis, preferably on formalin-fixed tissue as it is easier for the ophthalmic pathologist to review suspect material. Formalin-fixed tissue is also easier for the referring specialist as it rules out logistical problems surrounding fresh sampling and biases by not including sebaceous carcinoma in the differential diagnosis.

MiRNAs have been shown to play a role in the carcinogenesis of multiple conjunctival tumors [21,22] including OSCs, but to date no studies have been published showing the differences between OSCs and SCCCs for diagnostic purposes. To our knowledge no studies on miRNA expression in OSSNs are available.

The aim of this study is to identify miRNAs that may be of importance for tumorigenesis in OSCs and SCCCs, to identify miRNAs that may be of prognostic importance, and to identify differentiating miRNAs for OSCs versus SCCCs.

## 2. Results

### 2.1. Patient Characteristics

The patient cohort consisted of 55 patients that have been diagnosed with SCCC or OSC in the Rotterdam Eye Hospital and the Erasmus MC between the years 1990 and 2020. All histopathological slides have been reviewed by an experienced ophthalmic pathologist for correct diagnosis, and cases with equivocal histopathological diagnosis or insufficient remaining tissue for additional analysis have been excluded. The patients were divided in a discovery cohort and a validation cohort. The discovery cohort consisted of 18 SCCC patients, 8 male and 10 female. The median age at diagnosis was 50.5 years (range 36–89). The median follow-up of the SCCC patients in the discovery cohort was 39 months (6–120). Half of the patients with SCCCs in the discovery cohort developed at least one metastasis (*n* = 9). The discovery cohort consisted of 14 OSC patients, 7 male and 7 female. The median age at diagnosis was 75 years (range 48–91). The median follow-up of the OSC patients in the discovery cohort was 34 months (12–156). A healthy control cohort of 12 patients with normal histopathology had a median age of 75 (range 57–85). The information on recurrences, metastases, TNM-stage, and Muir –Torre syndrome are provided (Table 1).

The validation cohort consisted of 12 SCCC patients. Median age at diagnosis was 56.5 years (range 31–88). The validation cohort consisted of 11 OSC patients. Median age at diagnosis was 69 years (range 48–88). Clinical features of the validation cohort are given in Table 2.

### 2.2. MicroRNA Analysis

#### 2.2.1. MicroRNA Expression Differentiates Normal Epithelium from Tumor

**Figure 1:**
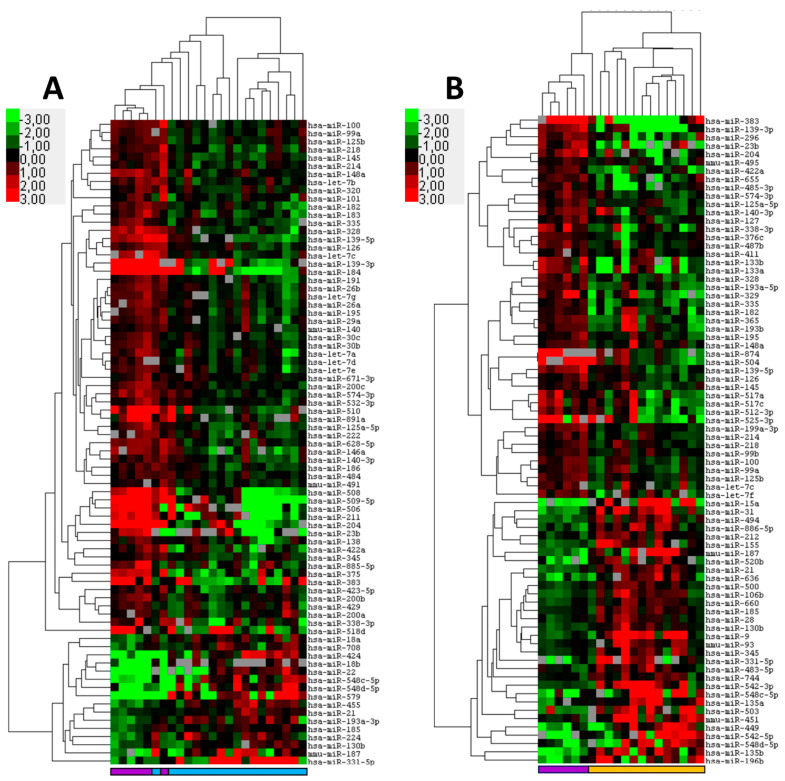
MicroRNA expression distinguishes squamous cell carcinoma of the conjunctiva (SCCC) and ocular sebaceous carcinoma (OSC) from healthy control tissues. Formalin fixed paraffin-embedded tumor samples and corresponding control tissue were subjected to miRNA profiling. Shown are heatmaps of supervised hierarchical clustering analyses based on significant (*p* < 0.05) differentially expressed miRNAs between tumor and its control tissue. (**A**) Cluster analysis of 18 SCCC and six controls consisting of conjunctival squamous epithelium of the eyelid based on 79 differentially expressed miRNAs. (**B**) Cluster analysis of 14 conjunctival OSC tumors and six Meibomian gland controls based on 78 differentially expressed miRNAs. In the heat map red indicates relative high expression, green relative low expression and grey designates missing expression values. The colored bars beneath the graph designate SCCC (light blue); OSC (orange) and controls (purple).

First, we investigated the differences in miRNA expression between normal epithelium and tumors using a well-characterized RT-PCR platform capable of detecting 377 human miRNAs. The comparison of normal conjunctival squamous epithelium and SCCCs revealed 79 differentially expressed miRNAs (*p* < 0.05) (Figure 1A; Supplementary Table S1). A supervised clustering based on the expression of these miRNAs almost completely distinguished between control and tumor tissue with a single SCCC sample clustering together with the controls. A close examination of the cluster tree showed two major groups, one consisting of controls and tumor samples, with the controls clustering together, and another group solely composed of SCCC samples. From the differentially expressed miRNAs, four were selected for further validation based on statistical parameters.

(*p* < 0.02; FDR < 5.6%) Signal intensity and shape of the amplification plot using individual RT-PCR assays. Three miRNAs, miR-383-5p, miR-424-5p, and miR-508-3p, but not miR-422a, were confirmed to be statistically differentially expressed between normal and tumor tissue (Supplementary Figure S1A). miR-424-5p (*p* ≤ 0.001) was found upregulated in SCCCs compared to the control and miR-508-3p (*p* ≤ 0.001) and miR-383-5p (*p* ≤ 0.01) were downregulated in SCCCs.

Similarly, the miRNA profiling of normal meibomian glands and OSCs identified 78 differentially expressed miRNAs that distinguish between control and OSC tissues (Figure 1B; Supplementary Table S2). Supervised cluster analysis demonstrated a virtual distinction between the controls and tumors with a single OSC sample clustering together with the controls. Five miRNAs were selected for validation using individual RT-PCR miRNA assays. All miRNAs were significantly differentially expressed, two miRNA miR-196-b-5p (*p* ≤ 0.05) and miR-636 (*p* ≤ 0.01) were upregulated in OSC when compared to the controls. Three miRNAs were downregulated in OSC miR-296-5p (*p* ≤ 0.05), miR-383-5p (*p* ≤ 0.01), and miR-422a (*p* ≤ 0.01) (Supplementary Figure S1B). It was noted that, compared to the control, miR-383-5p was downregulated in both OSCs and SCCCs, which may signify a common carcinogenic pathway between these two tumor types.

#### 2.2.2. No Prognostic microRNAs for Metastatic SCCC or Recurrent OSC

**Figure 2:**
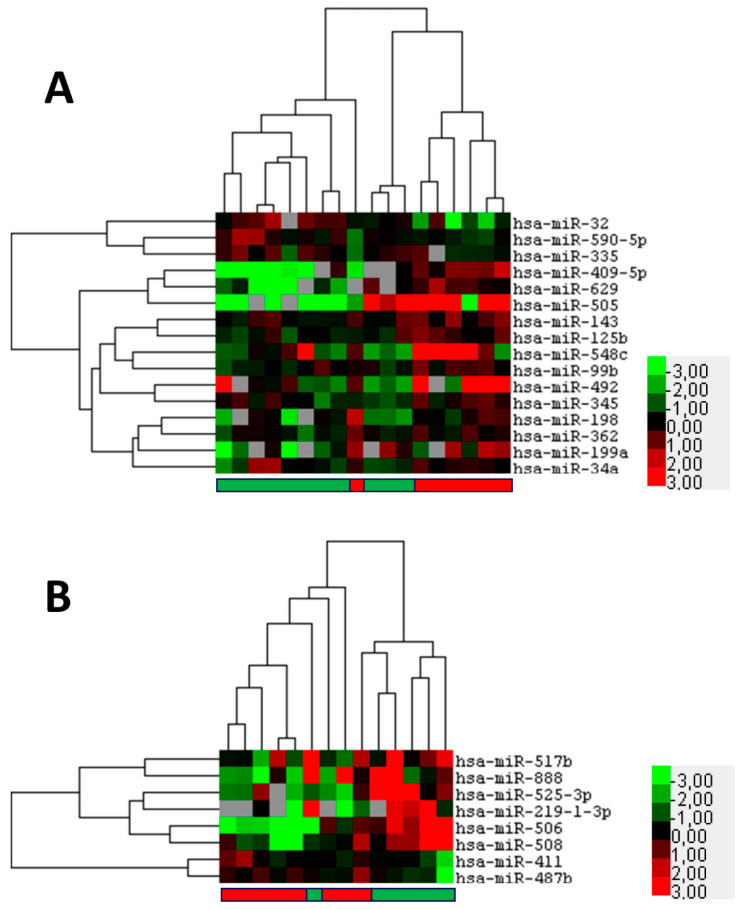
Potential prognostic microRNAs that indicate metastasis of squamous cell carcinoma of the conjunctiva (SCCC) or recurrence of ocular sebaceous carcinoma (OSC). Formalin fixed paraffin-embedded primary tumor samples were subjected to miRNA profiling. (**A**) Heatmap of supervised hierarchical clustering analyses based on 16 significant (*p* < 0.05) differentially expressed miRNAs between SCCC that do (red bar below) or do not metastasize (green bar below). (**B**) Heatmap of supervised hierarchical clustering analyses based on eight significant (*p* < 0.05) differentially expressed miRNAs between OSC that do (red bar below) or do not recur (green bar below). In the figure, red indicates relative high expression, green relative low expression, and grey designates missing expression values.

Next, it was examined whether miRNAs with prognostic significance could be identified for the two tumor types SCCC and OSC. A comparison of primary SCCC samples, using the miRNA expression data from the discovery set that eventually do or do not metastasize, revealed 16 differentially expressed miRNAs (*p* < 0.05; FDR 46.6–66.9%). A supervised cluster analysis based on the expression of these differentially expressed miRNAs showed that the majority of metastatic SCCC samples clustered away from the non-metastatic samples (Figure 2A; Supplementary Table S3). However, when a selection of miRNAs, miR-32; miR-130b; miR-199a; and miR-629 were validated using single RT-PCR assays, none of the miRNA showed a robust statistically significant differential expression by Mann–Whitney U testing (Supplementary Figure S2). For the OSC cohort metastatic disease was too rare to be analyzed; therefore, we chose to test for the recurrent disease instead. In OSC the miRNA expression profiles of cases showing recurrence were compared with non-recurrent cases highlighting eight miRNAs that are differentially expressed between the groups (*p* < 0.05; FDR 90.2%) (Figure 2B; Supplementary Table S4). A supervised clustering showed a clear tendency to separate OSC samples that recur from those that do not (Figure 2B). However, because of the small number and high FDR of the differentially expressed miRNA, we abstained from further validation. In conclusion, we were unable to indicate robust potential prognostic biomarkers for SCCC metastasis and OSC recurrence.

#### 2.2.3. Differentiating OSC from SCCC

**Figure 3:**
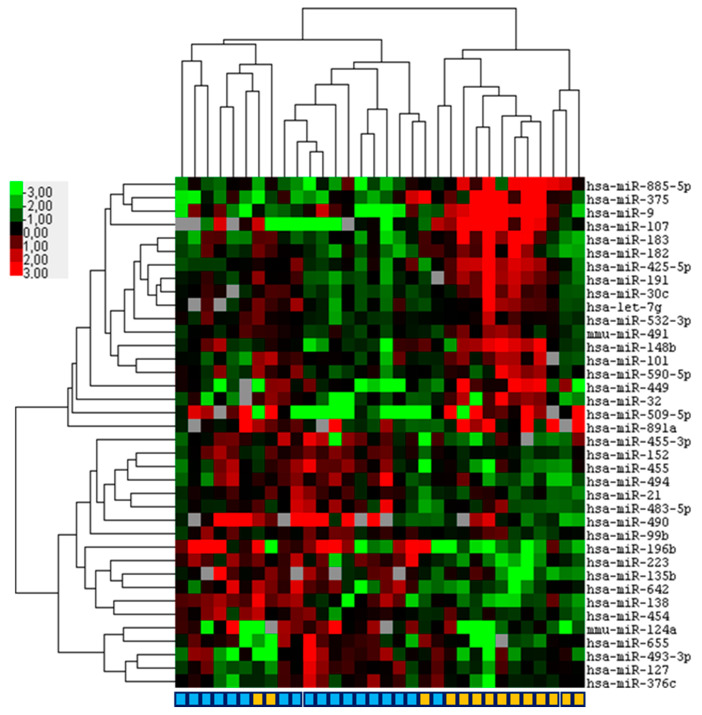
Hierarchical clustering based on miRNA expression virtually discriminates squamous cell carcinoma of the conjunctiva (SCCC) and ocular sebaceous carcinoma (OSC). Formalin fixed paraffin-embedded primary SCCC (*n* = 18) and OSC (*n* = 14) samples were subjected to miRNA profiling. Depicted is a heatmap of a supervised hierarchical clustering analyses based on 38 significant (*p* < 0.05) differentially expressed miRNAs between SCCC and OSC. In the figure, red indicates relative high expression, green relative low expression and grey designates missing expression values. In the color bar beneath, light blue indicates SCCC and orange OSC.

Finally, we investigated whether miRNA expression profiling can differentiate between OSC and SCCC. It was found that the expression of 38 differentially expressed miRNAs (*p* < 0.05; FDR (5.4–53.5%)) almost completely discriminated between OSC and SCCC with a misclustering of three OSC and one SCCC samples (Figure 3; Supplementary Table S5). One SCCC clustered within the OSC group, which was derived from a 77-year-old male who required exenteration and died of disease 36 months after diagnosis. Two OSC cases clustered within the SCCC group, one male and one female, aged 79 and 70, respectively. One case was completely resected without recurrence and alive at the last follow up, one case had a recurrence that required exenteration that was declined by the patient and the patient deceased of disease 72 months after diagnosis. All cases were reviewed for histopathological features that confirmed the original diagnosis.

Based on the expression level, statistical significance, and the shape of the amplification plots, four miRNAs were selected for validation using single RT-PCR assays in the discovery and validation cohort (Supplementary Figure S3). At least two, miR-107 and miR-196b-5p, of the selected miRNAs showed a robust statistically significant differential expression by Mann–Whitney U testing in both the discovery and validation cohort. MiR-107 was upregulated in OSC (*p* ≤ 0.005) and miR-196b-5p was upregulated in SCCC (*p* ≤ 0.05) (Supplementary Figure S3). One other miRNA, miR-493-3p, showed a trend for statistical significance in the discovery cohort, and was found significantly differentially expressed in the validation cohort (*p* ≤ 0.05) with an upregulation in SCCC compared to OSC (Supplementary Figure S3). It is interesting to find that although miR-196b-5p is upregulated in OSC when compared to normal sebaceous glands it is still significantly higher expressed in SCCC when compared to OSC.

**Figure 4:**
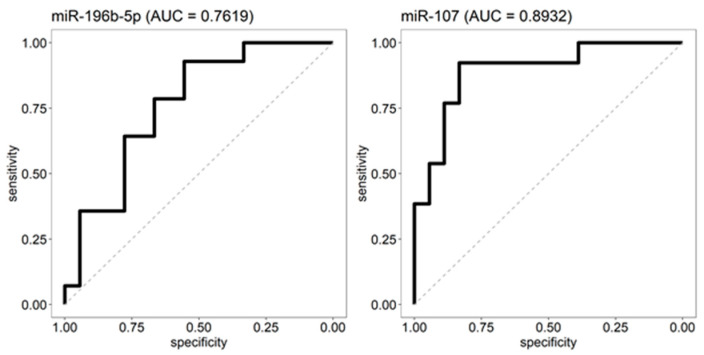
ROC analyses. The absolute expression levels of miR-196b-5p and miR-107 in the discovery cohort were used as input for a receiver operating characteristic (ROC) analysis to determine their validity as OSC and SCCC distinguishing biomarkers. For both miRNAs, a cut-off was chosen at which at least 90% of the OSC are categorized in the right group (90% sensitivity). For miR-196b-5p, the cut-off yielded a 93% sensitivity and 56% specificity, samples below the threshold are predicted as OSC. For miR-107 the cut-off yielded a 92% sensitivity and 83% specificity, samples above the threshold are considered OSC.

ROC analyses using the expression of miR-196b-5p and miR-107 in the discovery cohort were used to select thresholds at a 90% sensitivity to classify OSC. For miR-196b-5p, this threshold was 1172.258, yielding a 93% sensitivity with a 56% specificity (Figure 4). For miR-107, the threshold was 365.4, showing a 92% sensitivity and a 83% specificity (Figure 4). To validate the predictive power of the two miRs, expression levels were measured in 22 samples from the independent cohort (10 OSC and 12 SCCC cases). The samples were classified using both miRs and their respective thresholds (see methods) to obtain a predicted label as OSC or SCCC. Comparing the predicted with the true status showed a 90.0% sensitivity and 83.3% specificity to detect OSC (for OSC 9 out of 10 are predicted correctly, for SCCC this was 10 out of 12).

## 3. Discussion

In this study we have identified differentially expressed miRNAs for tumors versus control tissues in SCCCs and OSCs, respectively. No prognostic miRNAs could be reliably identified for either tumor type. Most importantly we demonstrate that the combined testing of miR196b-5p and miR-107 expression levels in tumor tissue, may be of additional use in routine diagnostics to discriminate OSCs from SCCCs.

For SCCC we validated three miRNAs to be differentially expressed between normal conjunctival squamous epithelium and SCCCs. We found miR-424-5p to be upregulated and miR-508-3p and miR-383-5p to be downregulated when compared to normal conjunctival squamous epithelium. There is no literature available on miRNA expression in OSSNs, including SCCCs for corroboration of these results. There are multiple reports on microRNA expression in pterygium of the conjunctiva, a condition that also involves epithelial-mesenchymal transition of epithelial cells. The MiRNAs miR-21, miR-143, miR-145, miR-182-5p, miR-183-5p, miR-184, miR-221 and miR-3175, have been described as upregulated and miR-221-3p andmiR-122 have been described to be downregulated in pterygium when compared to normal conjunctival epithelium of the limbal area. In our data set we find miR-21 slightly upregulated in the SCCC samples compared to the control, and miR-145, miR-182, miR-183 and miR-184 all downregulated in SCCC. MiR-143, miR-221 and miR-3175 were not designated as differentially expressed in our study.

For OSC we validated five miRNAs to be differentially expressed between normal meibomian gland epithelium and tumors. miR-196-b-5p and miR-636 were upregulated, whereas miR-296-5p, miR-383-5p and miR-422a were downregulated in OSC. Earlier studies on miRNA expression in OSC only show very limited overlap with our findings. This may be due to the different detection platforms that were used and a diverging scientific focus. In addition, these studies, however, suffered from low numbers of cases tested [23,24] and/or a lack of validation in independent patient cohorts [23,24,25,26,27]. The control tissues used also differed, varying from the normal meibomian gland [26] to tarsal plate tissue [24], sebaceous adenoma [23,27], and normal epidermis [25]. In addition to varying technical approaches, these factors explain the lack of correlation of findings between the different published studies and the current study.

It is interesting that although miR-196b-5p is upregulated in OSCs when compared to normal sebaceous gland epithelium, it is still significantly higher expressed in SCCCs when compared to OSCs. It may be hypothesized that miR-196-5p explains squamous differentiation seen in OSCs; a feature that may complicate correct diagnosis. This hypothesis may be supported by the fact that the upregulation of miR-196-5p is also described in oral squamous cell carcinomas that also arises in non-keratinizing mucosal epithelium [28]. In an earlier study on conjunctival melanoma, we have also observed miR-196-5p upregulated in melanoma versus benign nevi, corroborating the more general role of this microRNA in carcinogenesis [21]. Indeed, miR-196 may play a critical role in cancer pathogenesis by targeting several regulation molecules including HOXB8, HMGA2, and annexin A1 [29].

The downregulation of miR-383-5p in both SCCCs and OSCs indicates a common pathway of tumorigenesis. The downregulation of this miRNA has recently been implicated in head and neck squamous cell carcinomas. It was described to enhance cell proliferation and invasion, epithelial-mesenchymal transition (EMT), and tumor growth in this tumor type [30]. In a first attempt to obtain insight in the regulation of pathways by the deregulated miRNAs in OSCs and SCCCs, we performed a KEGG pathway analyses using DAVID and predicted miRNA targets by TargetScan as input (Supplementary Figures S4 and S5). Of interest is the fact that the MAPK signaling pathway and signaling pathways regulating the pluripotency of stem cells appear in the top 10 most significant pathways in both analyses. More effort is needed to establish the precise role of miRNAs in the carcinogenic processes of OSCs and SCCCs as more insight in the biology of these tumors may open up novel therapeutic strategies.

We were not able to identify prognostic miRNA expression patterns for either tumor. This may be due to small sample size of the discovery cohort. Otherwise, more complex (epi-) genetic regulation and external factors may play a role, requiring an alternative approach including big data analysis.

The histopathologic diagnoses of SCCCs and OSCs can be challenging, even for the experienced pathologist. OSCs can be recognized by infiltrating cells that have a finely vacuolated, frothy lipid containing cytoplasm. Mitotic activity is usually high and there is pronounced nuclear pleomorphisms. Some areas may, however, closely resemble squamous cell carcinoma. SCCCs are generally better differentiated, have more abundant eosinophilic cytoplasm without lipid vacuoles, and may show squamous eddy formations and keratin cysts. On the other hand, SCCCs may undergo lipoid degeneration. The problem of discerning poorly differentiated cases of OSCs from SCCCs is even more difficult. Immunohistochemical staining and conventional DNA based molecular studies do not solve this problem [11,15,16,17,31,32]. With this study we identified at least two discriminating microRNAs: miR-196b-5p and miR-107. We clearly demonstrated the power of these two miRNAs in predicting OSCs. Determining the expression levels of these two miRNAs in tumor tissue may be of additional use in routine diagnostics to discriminate benign from malignant conjunctival melanocytic lesions, especially in case the amount of tissue or the currently available techniques seem to be insufficient. In the future, the diagnosis may possibly be made based on liquid biopsies in blood or tear film, rendering the histopathologic features no longer applicable [33]. Additionally, miRNAs may be of interest with regard to targeted therapy [34,35].

## 4. Materials and Methods

### 4.1. Patients and Samples

For the primary analysis, a discovery set consisting of 14 conjunctival OSC tumors and 18 SCCC tumors was used. Furthermore, 6 samples of Meibomian glands were used as normal control tissues for OSCs and 6 samples of conjunctival squamous epithelium of the eyelid were used as control tissues for SCCCs. The control tissues were derived from non-diseased cosmetic eyelid surgical specimens. For an independent validation cohort, 11 cases of OSC and 12 cases of SCCC were used. The specimens were collected at the department of pathology of the Erasmus MC University Medical Center Rotterdam, the Netherlands. Clinical information and follow-up are provided for the discovery set in Table 1 and for the validation set in Table 2.

### 4.2. RNA Isolation

Hematoxylin-eosin-stained sections of formalin-fixed paraffin-embedded (FFPE) surgical resections were reviewed, and areas were selected containing at least 60% tumor cells and avoiding contamination with normal epithelium and inflammatory infiltrate. The FFPE tissue was cut, and total RNA was isolated by microdissection from 4 micrometer thick sections using the RecoverAll^TM^ Total Nucleic Acid Isolation Kit (Ambion/Life technologies) according to manufacturer’s protocol.

### 4.3. MicroRNA Expression Profiling

MiRNA expression profiles were determined using Taqman^®^ Low Density Array (TLDA) cards (A card v2.0, Applied Biosystems/ThermoFisher Scientific, Waltham, MA, USA). A cDNA pool was prepared using MegaplexTM Primers Human Pool (pool A v2.1, Applied Biosystems/ThermoFisher Scientific) and Taqman^®^ microRNA reverse Transcription kit (Applied Biosystems/Thermo Fisher). Next, the cDNA pool was amplified using MegaplexTM PreAmp Primers Human Pool (pool A v2.1) together with the Taqman^®^ PreAmp master-mix (Applied Biosystems/ThermoFisher Scientific). The resulting product was loaded in human miRNA A cards using Taqman^®^ Universal PCR Mix No AmpErase^®^ UNG (Applied biosystems/ThermoFisher Scientific) and run in a 7900HT Real-time PCR system (Applied biosystems/ThermoFisher Scientific). The expression of each miRNA in one sample was normalized to the median C_T_ value of all detectable miRNAs in that sample. The normalized relative expression was subsequently calculated for each miRNA and log transformed. A Mann–Whitney U test was performed to identify miRNAs that were differentially expressed between the groups. A two-sided *p*-value < 0.05 was considered as statistically significant. To adjust for multiple testing, a false discovery rate (FDR) was used. For hierarchical clustering analyses, the software program Cluster 3.0 was used followed by Java TreeView for visualization of the clustering results. The clustering was based on the uncentered correlation as a distance metric using average linkage.

### 4.4. Validation MicroRNA Expression

After profiling, amplification curves of differentially expressed miRNAs were inspected in the ThermoFisher Cloud App, and those exhibiting robust amplification were selected fur further investigation. Expression of these selected miRNAs was assessed by RT-PCR using commercially available Taqman^®^ MicroRNA assays (Applied Biosystems/ThermoFisher Scientific). In brief, 50 ng of isolated RNA was reverse transcribed using the Taqman^®^ MicroRNA reverse transcription kit (Applied Biosystems/ThermoFisher Scientific) and specific miRNA primers from the Taqman^®^ MicroRNA assays according to the manufacturer’s protocol. The resulting cDNA was pre-amplified and used in a quantitative real-time PCR (qPCR) using a miRNA specific primer/probe mixed together with the Taqman^®^ Universal PCR Mix No AmpErase^®^ UNG (Applied biosystems) in a QuantStudioTM 7 Flex Real-Tim PCR system (Applied Biosystems/ThermoFisher Scientific). qPCR data was analyzed using Quantstudio RT-PCR system software (version 1.3, Applied Biosystems). A standard dilution series of a cDNA sample-pool was included on every plate allowing absolute quantification of the miRNA expression. Differences in miRNA expression between the groups was evaluated by Mann–Whitney U tests, in which a two-sided *p*-value ≤ 0.05 was considered statistically significant and marked by a single asterisk (*). *p*-values ≤ 0.01 were marked by double asterisks (**) and ≤ 0.001 by triple asterisks (***). All statistical tests were performed with SPSS (IBM).

### 4.5. Predicting OSC Status Using ROC Analysis

Using the absolute expression levels of miR-196b-5p and miR-107 in our discovery cohort a receiver operating characteristic (ROC) analysis was performed to determine their validity as OSC and SCCC distinguishing biomarkers. For both miRNAs, a cut-off was chosen at which at least 90% of the OSCs are categorized in the right group (90% sensitivity). For miR-196b-5p, the cut-off was 1172.258 and samples below this threshold are predicted as OSCs, while for miR-107 the cut-off was 365.4 with samples above this level are considered as OSCs. Subsequently, expression levels of the samples in the independent validation cohort were measured and used to classify a sample according to the following steps: (1) Call a sample OSC when miR-196b-5p ≤ 1172.258, otherwise SCCC. (2) Call a sample OSC when miR-107 ≥ 365.4, otherwise SCCC. (3) Final call: only predict sample as OSC when both miR-196b-5p and miR-107 predict the sample as OSC.

## 5. Conclusions

Our findings indicate that deregulated miRNAs, identified by comparing tumor tissues with corresponding control tissues, may play a role in the tumorigenic processes in OSCs and SCCCs. Further studies are warranted to further decipher these functions. We were unable to identify robust prognostic miRNA biomarkers that predict metastasis in SCCCs and recurrence in OSCs emphasizing the clinical need for further research. Importantly, we provide evidence that miR-196b-5p and miR-107 can differentiate OSCs from SCCCs. The combined testing of miR-196b-5p and miR-107, may be of additional use in routine diagnostics to discriminate OSCs from SCCCs in conjunctival tumor lesions, in case the amount of tissue or the currently available techniques seem to be insufficient. A well-recognized diagnostic conundrum in ophthalmic pathology. Additionally, miRNAs may be of interest with regard to targeted therapy.

## Figures and Tables

**Table 1:** Patient characteristics of the discovery cohort.

		Controls	SCCC	OSC
Gender	m	6	8	7
	f	6	10	7
median age at diagnosis [range]		75 [59–85]	50.5 [36–89]	75 [48–91]
median follow up [range in months]			39 [6–120]	34 [12–156]
Muir–Torre Syndrome			0	0
Lynch Syndrome			1	0
TNM at diagnosis	T1		7	4
	T2		4	3
	T3		1	6
	T4		6	1
	N		6	2
	M		5	0
Follow up:				
	exenteration		6	5
	metastases		6	3
	recurrences		2	8
	death by disease		5	3
prognosis bad			7	8
prognosis good			11	6

**Table 2:** Patient characteristics of the validation cohort.

		SCCC	OSC
Gender	m	5	7
	f	7	4
median age at diagnosis [range]		56.5 [31–88]	69 [48–88]
median follow up [range in months]		24.6 [3–85]	36 [2–122]
Muir–Torre Syndrome		0	0
Lynch Syndrome		0	0
TNM at diagnosis	T1	6	0
	T2	4	7
	T3	1	3
	T4	1	1
	N	1	0
	M	0	0
Follow up:			
	exenteration	2	4
	metastases	0	1
	recurrences	5	4
	death by disease	0	1

## Data Availability

The miRNA expression data have been submitted to the Gene Expression Omnibus and are publicly accessible under accession number GSE201490.

## Supplementary Materials

The following supporting information can be downloaded at: https://www.mdpi.com/article/10.3390/ijms23094877/s1.
