# Supplementary material for: miR-196b-5p and miR-107 Expression Differentiates Ocular Sebaceous Carcinoma from Squamous Cell Carcinoma of the Conjunctiva
Source: Int J Mol Sci. 2022 Apr 28;23(9):4877. doi: 10.3390/ijms23094877 (PMC9102373; doi:10.3390/ijms23094877)
Supplement: Supplementary file 1 [file ijms-23-04877-s001.zip › ijms-1689183-supplementary R1 EW corrected file.pdf]

Supplemental Information

miR-196-5p and miR-107 expression differentiates ocular sebaceous carcinoma from squamous cell carcinoma of the conjunctiva

Ronald O.B. de Keizer, Anne L.M. Vriends, Gijsbert J. Hötte, Dion A. Paridaens, Erik A.C. Wiemer and Robert M. Verdijk

Table S1. Differentially expressed microRNAs between squamous cell carcinoma of the conjunctiva (SCCC) and corresponding healthy control tissue. Listed are 79 miRNAs ($p<0.05$) that are differentially expressed between SCCC (n=18) and control tissue (conjunctival squamous epithelium of the eyelid) (n=6) and that were used in the supervised hierarchical clustering (Figure 1A). p-values of Mann Whitney U test and false discovery rate p-values (FDR) are listed as well as the average expression level ratios of control/SCCC and SCCC/control.

miRNA	Control/SCCC	SCCC/Control	P-value	FDR
**hsa-miR-148a**	3.363924	0.297272	0.0001	0.001428
**hsa-miR-18b**	0.014008	71.38607	0.0001	0.001428
**hsa-miR-200c**	2.327974	0.429558	0.0001	0.002044
**hsa-miR-375**	7.901268	0.126562	0.0001	0.002044
**hsa-miR-100**	2.61338	0.382646	0.0001	0.003746
**hsa-miR-139-5p**	5.778269	0.173062	0.0001	0.003746
**hsa-miR-509-5p**	166.6427	0.006001	0.0001	0.004612
**hsa-miR-628-5p**	2.976465	0.335969	0.0001	0.004612
**hsa-miR-328**	4.363051	0.229197	0.0001	0.007177
**hsa-miR-26b**	2.492053	0.401276	0.0001	0.008889
**hsa-miR-182**	2.729625	0.366351	0.0001	0.010049
**hsa-miR-195**	2.550753	0.392041	0.0001	0.011027
**hsa-miR-146a**	4.466717	0.223878	0.0001	0.015449
**hsa-miR-508**	29.70985	0.033659	0.0001	0.01888
**hsa-miR-424**	0.029198	34.24947	0.001	0.007177
**hsa-miR-574-3p**	2.576914	0.388061	0.001	0.008889
**hsa-miR-200b**	2.302402	0.434329	0.001	0.008889
**hsa-miR-18a**	0.366928	2.725333	0.001	0.008889
**hsa-miR-191**	2.515353	0.397559	0.001	0.008965
**hsa-miR-99a**	2.095678	0.477173	0.001	0.01308
**hsa-miR-218**	2.675681	0.373737	0.001	0.016045
**hsa-miR-126**	2.198917	0.454769	0.001	0.017732
**hsa-let-7g**	2.405634	0.415691	0.001	0.019065
**hsa-miR-211**	44.39504	0.022525	0.001	0.086494
**hsa-let-7c**	4.16432	0.240135	0.002	0.007177
**hsa-miR-548c-5p**	0.069165	14.45814	0.002	0.007653
**hsa-miR-139-3p**	313.1079	0.003194	0.002	0.014824
**hsa-miR-30c**	2.109663	0.474009	0.002	0.015463
**hsa-miR-26a**	2.353042	0.424982	0.002	0.016045
**hsa-miR-335**	2.273675	0.439817	0.002	0.018827
**hsa-miR-29a**	2.186995	0.457248	0.002	0.04048
**hsa-miR-184**	88.17474	0.011341	0.003	0.016045
**hsa-miR-183**	2.797199	0.3575	0.003	0.023609
**hsa-miR-383**	30.73477	0.032536	0.003	0.05104
**hsa-miR-125a-5p**	2.482968	0.402744	0.004	0.016175
**hsa-miR-455**	0.325801	3.069354	0.004	0.017808
**hsa-miR-125b**	1.990835	0.502302	0.004	0.021217
**hsa-let-7b**	2.153847	0.464286	0.004	0.02871

**mmu-miR-491**	1.731597	0.577502	0.004	0.034014
**hsa-miR-186**	1.936481	0.516401	0.004	0.037334
**hsa-miR-224**	0.349602	2.860394	0.004	0.03969
**hsa-miR-204**	34.29999	0.029155	0.004	0.040969
**hsa-miR-423-5p**	2.23569	0.447289	0.004	0.054769
**hsa-miR-510**	23.70807	0.04218	0.005	0.037334
**hsa-miR-145**	2.20847	0.452802	0.006	0.027343
**hsa-miR-21**	0.535989	1.865711	0.006	0.034014
**hsa-miR-506**	28.81457	0.034705	0.006	0.047251
**hsa-let-7d**	1.748937	0.571776	0.006	0.064436
**hsa-miR-22**	0.002847	351.2929	0.008	0.008889
**mmu-miR-140**	1.560827	0.640686	0.008	0.095803
**hsa-miR-30b**	1.884525	0.530638	0.009	0.03043
**hsa-miR-140-3p**	2.0204	0.494952	0.009	0.053991
**hsa-let-7a**	2.526337	0.39583	0.009	0.15602
**hsa-miR-708**	0.45226	2.211119	0.012	0.081142
**hsa-miR-320**	2.033516	0.491759	0.015	0.03043
**hsa-miR-193a-3p**	0.39286	2.545437	0.015	0.075105
**hsa-miR-422a**	3.133185	0.319164	0.016	0.055552
**hsa-let-7**^{e}	1.915082	0.522171	0.016	0.13616
**hsa-miR-214**	1.907501	0.524246	0.018	0.052541
**hsa-miR-532-3p**	1.772962	0.564028	0.018	0.064469
**hsa-miR-101**	2.176836	0.459382	0.0185	0.053991
**hsa-miR-130b**	0.476517	2.09856	0.02	0.064469
**hsa-miR-579**	0.055099	18.14913	0.022	0.040969
**hsa-miR-345**	1.778152	0.562382	0.022	0.06593
**hsa-miR-548d-5p**	0.123125	8.121818	0.022	0.06593
**hsa-miR-429**	1.933766	0.517126	0.022	0.137874
**hsa-miR-185**	0.615875	1.623707	0.027	0.074389
**hsa-miR-23b**	33.44478	0.0299	0.027	0.218984
**hsa-miR-891a**	10.50865	0.09516	0.029	0.054769
**hsa-miR-338-3p**	4.614451	0.216711	0.03	0.265803
**hsa-miR-484**	1.471226	0.679705	0.033	0.125358
**hsa-miR-138**	2.2001	0.454525	0.033	0.181722
**hsa-miR-200a**	1.758164	0.568775	0.037	0.246984
**hsa-miR-222**	2.157112	0.463583	0.04	0.13616
**mmu-miR-187**	0.291636	3.42893	0.046	0.405157
**hsa-miR-331-5p**	0.05677	17.6149	0.047	0.064469
**hsa-miR-671-3p**	1.532939	0.652342	0.047	0.105328
**hsa-miR-885-5p**	3.546121	0.281998	0.047	0.115283
**hsa-miR-518d**	7.561453	0.13225	0.048	0.239144

Table S2. Differentially expressed microRNAs between ocular sebaceous carcinoma (OSC) and corresponding healthy control tissue. Listed are 78 miRNAs ($p<0.05$) that are differentially expressed between OSC (n=14) and control tissue (Meibomian glands) (n=6) and that were used in the supervised hierarchical clustering (Figure 1B). p-values of Mann Whitney U test and false discovery rate p-values (FDR) are listed as well as the average expression level ratios of control/OSC and OSC/control.

miRNA	Control/OSC	OSC/Control	P-value	FDR
**hsa-miR-139-5p**	3.726178	0.268371	0.0001	0.000301465
**hsa-miR-199a-3p**	2.496246	0.400602	0.0001	0.000301465
**hsa-miR-328**	2.918013	0.342699	0.0001	0.000301465
**hsa-miR-130b**	0.401121	2.493013	0.0001	0.000301465
**hsa-miR-376c**	3.114404	0.321089	0.0001	0.000301465
**hsa-miR-31**	0.139595	7.163568	0.001	0.000301465
**hsa-miR-383**	75.38322	0.013266	0.001	0.000301465
**hsa-miR-139-3p**	106.1066	0.009424	0.001	0.000301465
**hsa-miR-15a**	0.010381	96.32702	0.001	0.000301465
**hsa-miR-204**	13.83021	0.072305	0.001	0.000301465
**hsa-miR-487b**	3.040194	0.328926	0.001	0.000301465
**hsa-miR-125b**	2.044631	0.489086	0.002	0.000301465
**hsa-miR-21**	0.351752	2.842911	0.002	0.000301465
**hsa-miR-494**	0.297415	3.362309	0.002	0.000301465
**hsa-miR-574-3p**	2.222661	0.449911	0.002	0.000301465
**hsa-miR-193a-5p**	2.750874	0.363521	0.002	0.000301465
**hsa-miR-335**	2.730895	0.36618	0.002	0.000301465
**hsa-miR-296**	3.84469	0.260099	0.002	0.000301465
**hsa-miR-182**	2.259389	0.442598	0.002	0.000301465
**hsa-miR-100**	2.336035	0.428076	0.003	0.000301465
**hsa-miR-886-5p**	0.318795	3.136816	0.003	0.000301465
**hsa-miR-338-3p**	4.648259	0.215134	0.003	0.000301465
**hsa-miR-874**	106.1133	0.009424	0.005	0.000301465
**hsa-miR-504**	89.59001	0.011162	0.005	0.005102499
**hsa-miR-214**	1.954108	0.511743	0.005	0.02594702
**hsa-miR-127**	2.028345	0.493013	0.005	0.02594702
**mmu-miR-187**	0.057611	17.35784	0.005	0.02594702
**hsa-miR-195**	1.901813	0.525814	0.005	0.02594702
**hsa-miR-520b**	0.075829	13.18763	0.006	0.02594702
**hsa-miR-125a-5p**	2.036363	0.491072	0.006	0.062187074
**hsa-miR-500**	0.502096	1.991649	0.006	0.071335425
**hsa-miR-636**	0.255577	3.912718	0.007	0.074872408
**hsa-miR-106b**	0.350331	2.854446	0.009	0.074872408
**hsa-miR-655**	4.508368	0.22181	0.009	0.074872408
**hsa-miR-422a**	3.84747	0.259911	0.009	0.074872408
**hsa-miR-485-3p**	4.506542	0.2219	0.009	0.074872408
**hsa-miR-503**	0.128631	7.774191	0.01	0.074872408
**hsa-miR-212**	0.499498	2.002009	0.012	0.074872408
**hsa-miR-329**	5.324067	0.187826	0.012	0.100211609
**hsa-miR-660**	0.534102	1.872301	0.012	0.100211609
**hsa-miR-155**	0.285607	3.501309	0.012	0.100211609
**hsa-miR-135b**	0.141648	7.059741	0.012	0.100211609
**hsa-miR-185**	0.558834	1.789439	0.015	0.100211609
**hsa-miR-140-3p**	2.73765	0.365277	0.015	0.100211609
**hsa-miR-542-3p**	0.113813	8.786305	0.015	0.100211609
**hsa-miR-449**	0.114493	8.734165	0.015	0.100211609
**hsa-miR-148a**	1.602759	0.623924	0.015	0.100211609
**hsa-miR-196b**	0.058826	16.99936	0.02	0.100211609

**hsa-miR-126**	1.850234	0.540472	0.02	0.109573987
**hsa-miR-483-5p**	0.516552	1.935915	0.02	0.109573987
**hsa-miR-365**	2.564304	0.389969	0.02	0.109573987
**hsa-miR-193b**	2.117824	0.472183	0.02	0.109573987
**mmu-miR-495**	2.656914	0.376376	0.02	0.109573987
**hsa-miR-133b**	16.64768	0.060068	0.022	0.109573987
**hsa-miR-542-5p**	0.073944	13.52377	0.023	0.109573987
**hsa-miR-9**	0.131343	7.613652	0.026	0.143452639
**hsa-miR-28**	0.575362	1.738035	0.026	0.145555496
**hsa-miR-345**	0.467307	2.139922	0.026	0.145555496
**hsa-miR-548c-5p**	0.106316	9.405941	0.029	0.145555496
**hsa-miR-331-5p**	0.045249	22.09972	0.03	0.153083206
**hsa-let-7f**	3.506015	0.285224	0.032	0.163268208
**hsa-miR-517a**	3.037029	0.329269	0.033	0.163268208
**hsa-miR-99a**	1.978096	0.505537	0.033	0.163268208
**hsa-miR-133a**	7.441203	0.134387	0.033	0.163268208
**hsa-miR-744**	0.595053	1.680523	0.033	0.163268208
**hsa-miR-218**	1.544023	0.647659	0.033	0.163268208
**hsa-miR-517c**	2.756074	0.362835	0.033	0.163268208
**hsa-miR-145**	1.65455	0.604394	0.033	0.163268208
**hsa-miR-135a**	0.318652	3.138217	0.034	0.163268208
**mmu-miR-451**	0.348596	2.868649	0.036	0.163268208
**mmu-miR-93**	0.436937	2.288659	0.036	0.163268208
**hsa-miR-23b**	41.3399	0.02419	0.037	0.163268208
**hsa-miR-99b**	1.489505	0.671364	0.041	0.163268208
**hsa-miR-512-3p**	2.623971	0.381102	0.041	0.163268208
**hsa-miR-411**	1.494263	0.669226	0.044	0.163268208
**hsa-miR-525-3p**	10.71605	0.093318	0.046	0.163268208
**hsa-miR-548d-5p**	0.169179	5.910906	0.046	0.183454131
**hsa-let-7c**	1.861897	0.537087	0.046	0.183454131

Table S3. Differentially expressed microRNAs between primary squamous cell carcinoma of the conjunctiva that metastasize ($SCCC_m$) and those that do not (SCCC-). Listed are 16 miRNAs ($p<0.05$) that are differentially expressed between primary SCCC (n=18) that eventually display metastases ($SCCC_m$) and those that did not metastasize in the time-frame of the follow-up (SCCC-) and that were used in the supervised hierarchical clustering (Figure 2A). p-values of Mann Whitney U test and false discovery rate p-values (FDR) are listed as well as the average expression level ratios of SCCC-/$SCCC_m$ and $SCCC_m$/SCCC-

miRNA	SCCC-/$SCCC_m$	$SCCC_m$/SCCC-	P- value	FDR
**hsa-miR-409-5p**	0.087115	11.47902	0.002	0.468
**hsa-miR-590-5p**	1.879544	0.532044	0.004	0.468
**hsa-miR-198**	0.375235	2.664998	0.007	0.468
**hsa-miR-32**	5.346134	0.187051	0.007	0.5482
**hsa-miR-143**	0.612528	1.632578	0.008	0.5482
**hsa-miR-629**	0.129307	7.733511	0.008	0.5482
**hsa-miR-548c**	0.102292	9.775917	0.015	0.468
**hsa-miR-99b**	0.61549	1.624722	0.015	0.5157
**hsa-miR-125b**	0.60897	1.642116	0.027	0.5482
**hsa-miR-199a**	0.179127	5.582624	0.029	0.669
**hsa-miR-505**	0.104684	9.552585	0.031	0.669
**hsa-miR-362**	0.636025	1.572266	0.035	0.5482
**hsa-miR-345**	0.648909	1.541048	0.044	0.669
**hsa-miR-34a**	0.667004	1.499241	0.044	0.669
**hsa-miR-335**	1.617858	0.618101	0.048	0.669

Table S4. Differentially expressed microRNAs between ocular sebaceous carcinoma that recur (OSC_R) and those that do not recur (OSC-). Listed are 8 miRNAs ($p<0.05$) that are differentially expressed between primary OSC (n=14) that eventually recur (OSC_R) and those that did not recur in the time-frame of the follow-up (OSC-) and that were used in the supervised hierarchical clustering (Figure 2B). p-values of Mann Whitney U test and false discovery rate p-values (FDR) are listed as well as the average expression level ratios of OSC-/OSC_R and OSC_R/OSC-.

miRNA	OSC-/OSC_R	OSC_R/OSC-	P-value	FDR
**hsa-miR-525-3p**	7,556668	0,132333	0,022	0,901513
**hsa-miR-219-1-3p**	21,36382	0,046808	0,032	0,901513
**hsa-miR-517b**	5,566092	0,179659	0,043	0,901513
**hsa-miR-888**	6,980908	0,143248	0,043	0,901513
**hsa-miR-506**	12,20891	0,081907	0,043	0,901513
**hsa-miR-508**	13,01921	0,07681	0,043	0,901513
**hsa-miR-411**	0,551286	1,81394	0,043	0,901513
**hsa-miR-487b**	0,336554	2,971289	0,043	0,901513

Table S5. Differentially expressed microRNAs between squamous cell carcinoma of the conjunctiva (SCCC) and ocular sebaceous carcinoma (OSC). Listed are 38 miRNAs ($p<0.05$) that are differentially expressed between SCCC ($n=18$) and OSC ($n=14$) and that were used in the supervised hierarchical clustering (Figure 3). p-values of Mann Whitney U test and false discovery rate p-values (FDR) are listed as well as the average expression level ratios of control/SCCC and SCCC/control.

miRNA	OSC/SCCC	SCCC/OSC	P-value	FDR
**hsa-miR-152**	0.549082	1.821222	0.0001	0.05408456
**hsa-miR-885-5p**	6.304642	0.158613	0.001	0.05408456
**hsa-miR-191**	2.647099	0.377772	0.001	0.055200656
**hsa-miR-223**	0.357044	2.800774	0.001	0.055200656
**mmu-miR-124a**	0.1435	6.968617	0.001	0.153161901
**hsa-miR-455**	0.333646	2.997191	0.002	0.055200656
**hsa-miR-183**	3.464555	0.288637	0.002	0.056371921
**hsa-miR-107**	17.86865	0.055964	0.002	0.101364558
**hsa-miR-375**	4.561179	0.219242	0.004	0.100487348
**hsa-miR-9**	7.334788	0.136337	0.005	0.071816105
**hsa-miR-455-3p**	0.411753	2.428641	0.005	0.153161901
**hsa-miR-425-5p**	2.421695	0.412934	0.007	0.133361292
**hsa-miR-196b**	0.152276	6.567035	0.007	0.135762401
**hsa-miR-493-3p**	0.314701	3.177615	0.009	0.357218414
**hsa-miR-494**	0.448891	2.227713	0.014	0.135762401
**hsa-miR-138**	0.463725	2.156453	0.014	0.294185387
**hsa-miR-21**	0.599696	1.667511	0.016	0.144977075
**hsa-miR-30c**	1.847739	0.541202	0.017	0.176456266
**hsa-miR-101**	2.070469	0.482982	0.018	0.118185238
**hsa-miR-148b**	2.851371	0.350708	0.02	0.231117144
**hsa-miR-182**	2.034129	0.491611	0.02	0.283798459
**hsa-miR-99b**	0.712166	1.404166	0.02	0.29024011
**hsa-miR-590-5p**	1.703094	0.587167	0.022	0.283798459
**hsa-miR-655**	0.387705	2.579283	0.022	0.379726354
**hsa-miR-32**	2.279261	0.438739	0.023	0.560289283
**hsa-miR-135b**	0.241916	4.133674	0.024	0.29024011
**hsa-miR-642**	0.510223	1.959926	0.025	0.439894732
**hsa-miR-449**	3.217069	0.310842	0.026	0.446197609
**hsa-miR-532-3p**	1.386819	0.721075	0.027	0.357218414
**hsa-miR-509-5p**	7.775498	0.128609	0.031	0.357218414
**hsa-miR-490**	0.089071	11.22701	0.033	0.105338384
**mmu-miR-491**	1.478838	0.676206	0.034	0.276178304
**hsa-miR-376c**	0.470283	2.126379	0.034	0.456599223
**hsa-miR-483-5p**	0.592341	1.688216	0.037	0.357218414
**hsa-miR-891a**	4.350374	0.229865	0.041	0.357218414
**hsa-let-7g**	1.605626	0.62281	0.047	0.379726354
**hsa-miR-127**	0.626917	1.595108	0.049	0.343479638
**hsa-miR-454**	0.723781	1.381634	0.049	0.535630419

Figure S1

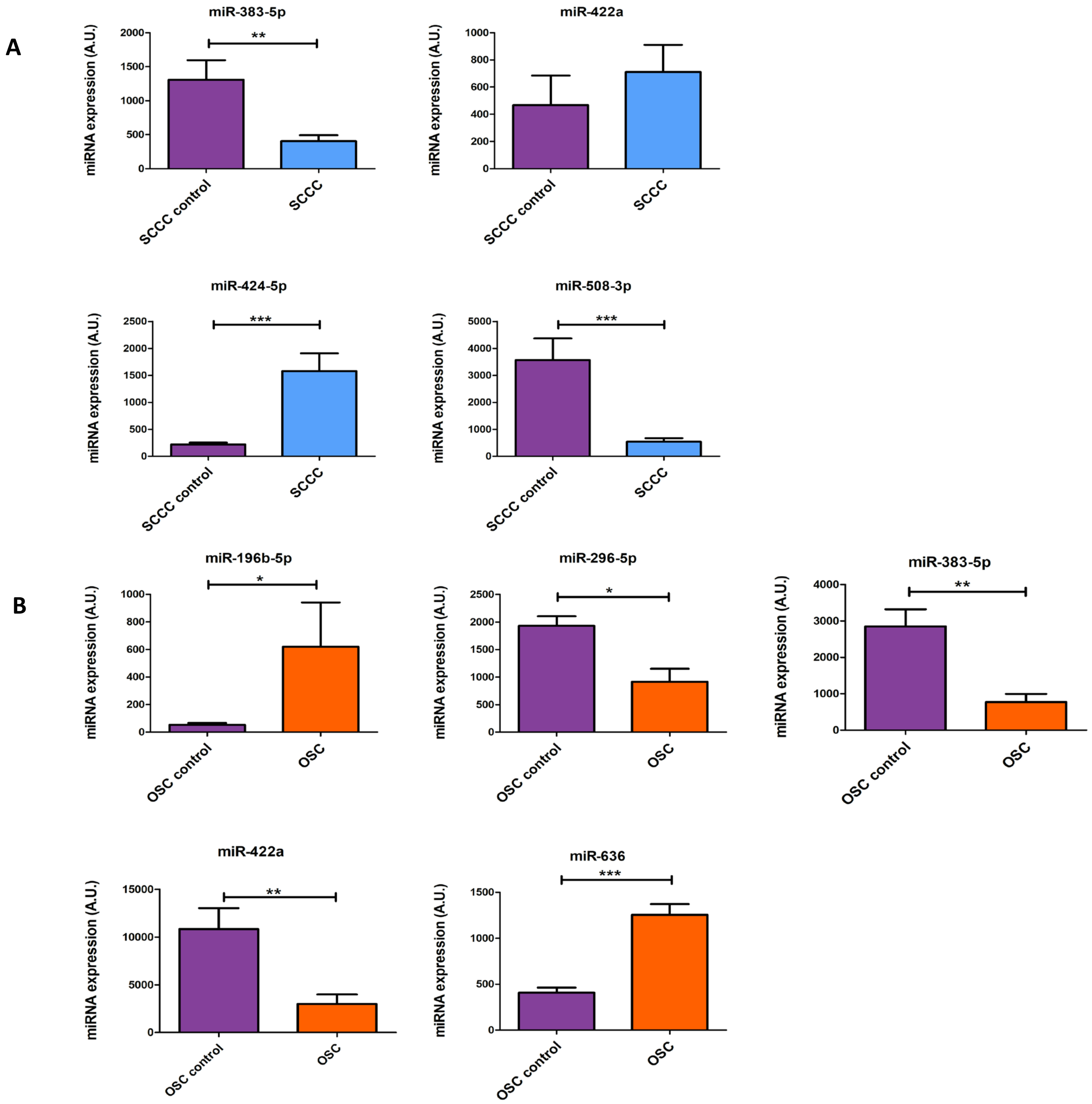

Figure S1. Quantitative RT-PCR verification of selected differentially expressed microRNAs in squamous cell carcinoma of the conjunctiva (SCCC) and ocular sebaceous carcinoma (OSC) and their corresponding control tissues. (A) A standardized RT-PCR procedure using individual assays was used to verify the expression of selected miRNAs found differentially expressed between SCCC and healthy control tissue (conjunctival squamous epithelium of the eyelid). The expression of miR-383-5p, miR-422a, miR-424-5p and miR-508-3p was determined in the original discovery cohort consisting of 18 SCCC and 6 control samples. (B) A standardized RT-PCR procedure using individual assays was used to verify the expression of selected miRNAs found differentially expressed between OSC and healthy control tissue (Meibomian glands). The expression of miR-196b-5p, miR-296-5p, miR-383-5p, miR-422a and miR-636 was determined in the original discovery cohort consisting of 14 OSC and 6 control samples. A standard dilution series of a cDNA samples-pool was used to determine absolute quantification of the miRNA expression. Depicted are average values ± S.D. Asterisks indicate statistically significant differences * $p<0.05$; ** $p<0.005$ and *** $p<0.0005$ (Mann Whitney U test)

Figure S2

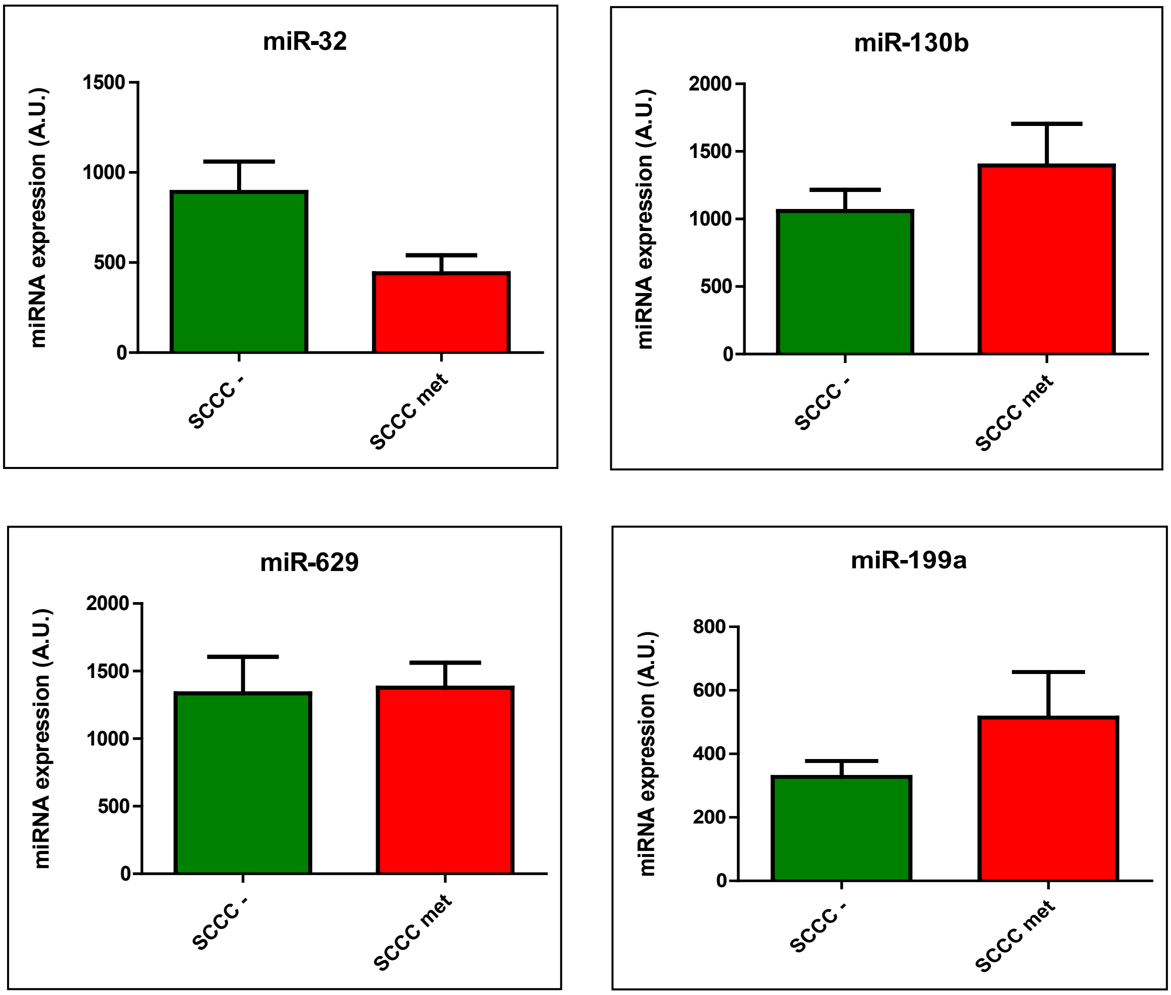

Figure S2. Quantitative RT-PCR verification of selected differentially expressed microRNAs in squamous cell carcinoma of the conjunctiva that metastasize ($SCCC_{met}$) and those that do not (SCCC-). A standardized RT-PCR procedure using individual assays was used to verify the expression of selected miRNAs found differentially expressed between primary SCCC (n=18) that eventually display metastases ($SCCC_{met}$) and those that did not metastasize in the time-frame of the follow-up (SCCC-) and that were used in the supervised hierarchical clustering (Figure 2A).

Figure S3

A

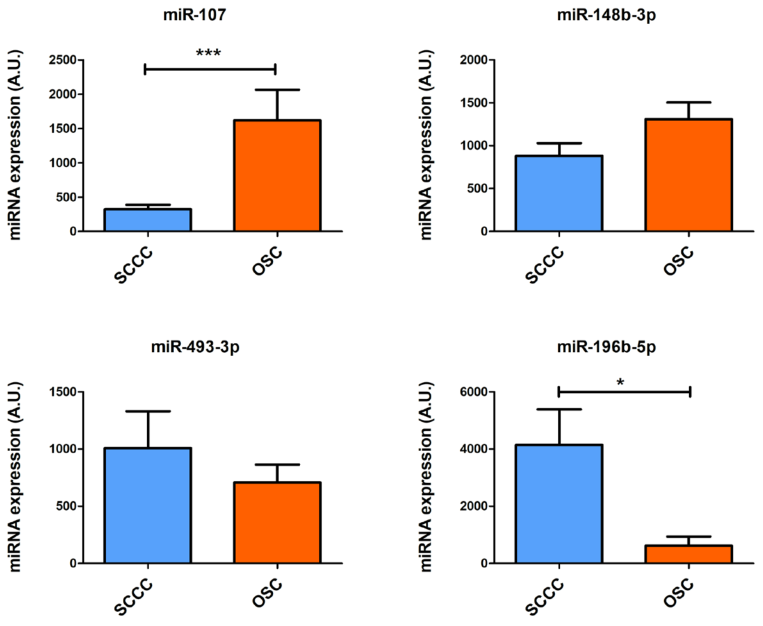

B

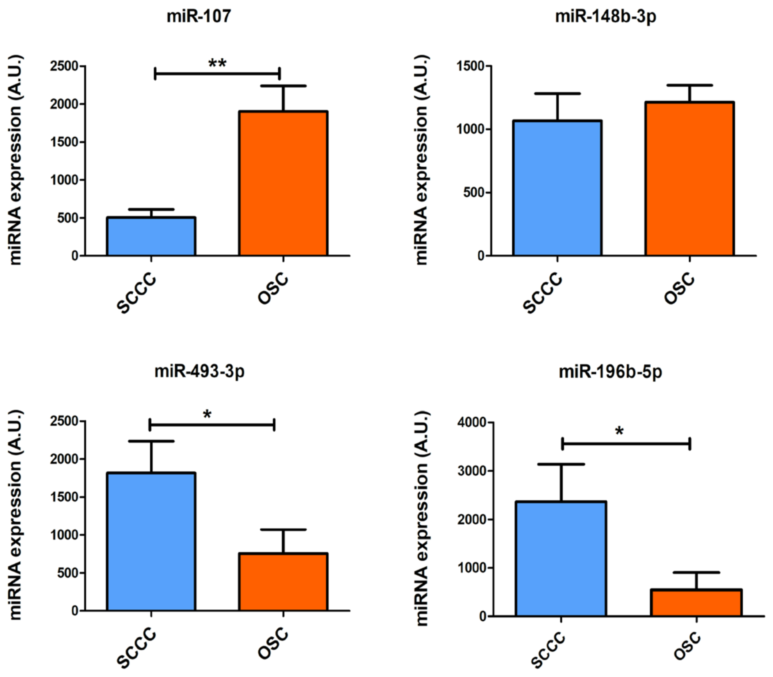

Figure S3. Quantitative RT-PCR verification of selected differentially expressed microRNAs in squamous cell carcinoma of the conjunctiva (SCCC) and ocular sebaceous carcinoma (OSC). A standardized RT-PCR procedure using individual assays was used to verify the expression of selected miRNAs found differentially expressed between SCCC and OSC (see Figure 3). The expression of miR-107, miR-148-3p, miR-196b-5p and miR-493-3p was determined in (A) the original discovery cohort consisting of 18 SCCC and 14 OSC samples and (B) an independent validation cohort consisting of 12 SCCC and 11 OSC samples. A standard dilution series of a cDNA samples-pool was used to determine absolute quantification of the miRNA expression. Depicted are average values ± S.D. Asterisks indicate statistically significant differences * $p<0.05$; ** $p<0.005$ and *** $p<0.0005$ (Mann Whitney U test).

Figure S4

Sublist	Category	Term	RT	Genes	Count	%	P-Value	Benjamini
	KEGG_PATHWAY	MAPK signaling pathway	RT		53	3.0	2.2E-6	7.3E-4
	KEGG_PATHWAY	Proteoglycans in cancer	RT		37	2.1	9.6E-5	1.6E-2
	KEGG_PATHWAY	Endocytosis	RT		42	2.4	1.7E-4	1.7E-2
	KEGG_PATHWAY	Relaxin signaling pathway	RT		26	1.5	2.3E-4	1.7E-2
	KEGG_PATHWAY	Ras signaling pathway	RT		39	2.2	2.8E-4	1.7E-2
	KEGG_PATHWAY	Oxytocin signaling pathway	RT		29	1.6	3.1E-4	1.7E-2
	KEGG_PATHWAY	Colorectal cancer	RT		19	1.1	6.6E-4	3.1E-2
	KEGG_PATHWAY	Signaling pathways regulating pluripotency of stem cells	RT		26	1.5	1.2E-3	3.5E-2
	KEGG_PATHWAY	Adipocytokine signaling pathway	RT		16	0.9	1.2E-3	3.5E-2
	KEGG_PATHWAY	Chronic myeloid leukemia	RT		17	1.0	1.2E-3	3.5E-2
	KEGG_PATHWAY	Human cytomegalovirus infection	RT		36	2.0	1.2E-3	3.5E-2
	KEGG_PATHWAY	Focal adhesion	RT		33	1.9	1.3E-3	3.5E-2
	KEGG_PATHWAY	Yersinia infection	RT		25	1.4	1.4E-3	3.5E-2
	KEGG_PATHWAY	Pathways in cancer	RT		69	3.9	2.5E-3	5.7E-2
	KEGG_PATHWAY	Rap1 signaling pathway	RT		33	1.9	2.8E-3	5.7E-2
	KEGG_PATHWAY	Prostate cancer	RT		19	1.1	2.8E-3	5.7E-2
	KEGG_PATHWAY	Kaposi sarcoma-associated herpesvirus infection	RT		31	1.8	3.0E-3	5.7E-2
	KEGG_PATHWAY	Viral carcinogenesis	RT		32	1.8	3.3E-3	5.8E-2
	KEGG_PATHWAY	FoxO signaling pathway	RT		23	1.3	3.7E-3	5.8E-2
	KEGG_PATHWAY	Prolactin signaling pathway	RT		15	0.9	4.0E-3	5.8E-2
	KEGG_PATHWAY	ErbB signaling pathway	RT		17	1.0	4.1E-3	5.8E-2
	KEGG_PATHWAY	Dopaminergic synapse	RT		23	1.3	4.1E-3	5.8E-2
	KEGG_PATHWAY	Hedgehog signaling pathway	RT		13	0.7	4.1E-3	5.8E-2
	KEGG_PATHWAY	Human papillomavirus infection	RT		46	2.6	4.4E-3	5.8E-2
	KEGG_PATHWAY	Autophagy - animal	RT		24	1.4	4.5E-3	5.8E-2
	KEGG_PATHWAY	Melanoma	RT		15	0.9	5.2E-3	6.4E-2
	KEGG_PATHWAY	Neurotrophin signaling pathway	RT		21	1.2	5.5E-3	6.4E-2
	KEGG_PATHWAY	Growth hormone synthesis, secretion and action	RT		21	1.2	5.5E-3	6.4E-2
	KEGG_PATHWAY	Insulin signaling pathway	RT		23	1.3	6.5E-3	7.3E-2
	KEGG_PATHWAY	Endocrine resistance	RT		18	1.0	7.3E-3	7.9E-2
	KEGG_PATHWAY	Glioma	RT		15	0.9	7.5E-3	7.9E-2
	KEGG_PATHWAY	Adherens junction	RT		14	0.8	1.1E-2	1.2E-1
	KEGG_PATHWAY	Osteoclast differentiation	RT		21	1.2	1.2E-2	1.2E-1
	KEGG_PATHWAY	AMPK signaling pathway	RT		20	1.1	1.3E-2	1.2E-1
	KEGG_PATHWAY	T cell receptor signaling pathway	RT		18	1.0	1.3E-2	1.2E-1
	KEGG_PATHWAY	Estrogen signaling pathway	RT		22	1.3	1.4E-2	1.3E-1
	KEGG_PATHWAY	Platelet activation	RT		20	1.1	1.8E-2	1.5E-1
	KEGG_PATHWAY	Wnt signaling pathway	RT		25	1.4	1.8E-2	1.5E-1
	KEGG_PATHWAY	Adrenergic signaling in cardiomyocytes	RT		23	1.3	1.8E-2	1.5E-1
	KEGG_PATHWAY	Salmonella infection	RT		34	1.9	1.9E-2	1.6E-1
	KEGG_PATHWAY	Pancreatic cancer	RT		14	0.8	2.0E-2	1.6E-1
	KEGG_PATHWAY	Tight junction	RT		25	1.4	2.0E-2	1.6E-1
	KEGG_PATHWAY	Melanogenesis	RT		17	1.0	2.1E-2	1.6E-1
	KEGG_PATHWAY	PI3K-Akt signaling pathway	RT		45	2.6	2.2E-2	1.7E-1
	KEGG_PATHWAY	Sphingolipid signaling pathway	RT		19	1.1	2.3E-2	1.7E-1
	KEGG_PATHWAY	TGF-beta signaling pathway	RT		16	0.9	2.3E-2	1.7E-1
	KEGG_PATHWAY	Longevity regulating pathway - multiple species	RT		12	0.7	2.4E-2	1.7E-1
	KEGG_PATHWAY	Toxoplasmosis	RT		18	1.0	2.6E-2	1.7E-1
	KEGG_PATHWAY	EGFR tyrosine kinase inhibitor resistance	RT		14	0.8	2.7E-2	1.7E-1
	KEGG_PATHWAY	Breast cancer	RT		22	1.3	2.7E-2	1.7E-1

Figure S4. KEGG pathway analysis using predicted gene targets of deregulated microRNAs in OSC. The predicted gene targets of miR-196b-5p, miR-296-5p, miR-383-5p, miR-422a and miR-636, all deregulated in OSC versus healthy control tissue, were downloaded from TargetScan (release 8.0; https://www.targetscan.org). The genes were subsequently used as input for the database for annotation, visualization and integrated discovery (DAVID- 2021 update; https://david.ncifcrf.gov) for a KEGG pathways analysis. Listed are the top 50 most significant pathways.

Figure S5

Sublist	Category	Term	RT	Genes	Count	%	P-Value	Benjamini
	KEGG_PATHWAY	Breast cancer	RT		36	1.8	5.0E-7	7.4E-5
	KEGG_PATHWAY	Signaling pathways regulating pluripotency of stem cells	RT		35	1.8	7.6E-7	7.4E-5
	KEGG_PATHWAY	Pathways in cancer	RT		88	4.5	7.9E-7	7.4E-5
	KEGG_PATHWAY	Neurotrophin signaling pathway	RT		31	1.6	9.1E-7	7.4E-5
	KEGG_PATHWAY	Prolactin signaling pathway	RT		21	1.1	8.0E-6	5.2E-4
	KEGG_PATHWAY	Gastric cancer	RT		33	1.7	1.6E-5	7.6E-4
	KEGG_PATHWAY	Proteoglycans in cancer	RT		41	2.1	1.7E-5	7.6E-4
	KEGG_PATHWAY	Hippo signaling pathway	RT		34	1.7	1.9E-5	7.6E-4
	KEGG_PATHWAY	MAPK signaling pathway	RT		52	2.7	3.7E-5	1.3E-3
	KEGG_PATHWAY	mTOR signaling pathway	RT		33	1.7	4.2E-5	1.3E-3
	KEGG_PATHWAY	Insulin signaling pathway	RT		30	1.5	5.2E-5	1.5E-3
	KEGG_PATHWAY	Measles	RT		30	1.5	6.8E-5	1.8E-3
	KEGG_PATHWAY	Chronic myeloid leukemia	RT		20	1.0	1.0E-4	2.5E-3
	KEGG_PATHWAY	Human papillomavirus infection	RT		55	2.8	1.2E-4	2.8E-3
	KEGG_PATHWAY	Renal cell carcinoma	RT		18	0.9	2.8E-4	6.1E-3
	KEGG_PATHWAY	Pancreatic cancer	RT		19	1.0	3.2E-4	6.5E-3
	KEGG_PATHWAY	Wnt signaling pathway	RT		32	1.6	3.7E-4	6.8E-3
	KEGG_PATHWAY	PI3K-Akt signaling pathway	RT		56	2.9	3.8E-4	6.8E-3
	KEGG_PATHWAY	Hepatitis B	RT		31	1.6	4.7E-4	8.1E-3
	KEGG_PATHWAY	p53 signaling pathway	RT		18	0.9	5.7E-4	9.3E-3
	KEGG_PATHWAY	Acute myeloid leukemia	RT		17	0.9	6.2E-4	9.5E-3
	KEGG_PATHWAY	Cellular senescence	RT		29	1.5	1.2E-3	1.7E-2
	KEGG_PATHWAY	AMPK signaling pathway	RT		24	1.2	1.3E-3	1.7E-2
	KEGG_PATHWAY	Hepatitis C	RT		29	1.5	1.3E-3	1.7E-2
	KEGG_PATHWAY	Small cell lung cancer	RT		20	1.0	1.3E-3	1.7E-2
	KEGG_PATHWAY	Melanoma	RT		17	0.9	1.4E-3	1.8E-2
	KEGG_PATHWAY	Colorectal cancer	RT		19	1.0	1.5E-3	1.8E-2
	KEGG_PATHWAY	Hepatocellular carcinoma	RT		30	1.5	1.8E-3	2.1E-2
	KEGG_PATHWAY	FoxO signaling pathway	RT		25	1.3	2.0E-3	2.2E-2
	KEGG_PATHWAY	Salmonella infection	RT		40	2.0	2.2E-3	2.4E-2
	KEGG_PATHWAY	Prostate cancer	RT		20	1.0	2.6E-3	2.7E-2
	KEGG_PATHWAY	Sphingolipid signaling pathway	RT		23	1.2	2.6E-3	2.7E-2
	KEGG_PATHWAY	Central carbon metabolism in cancer	RT		16	0.8	2.9E-3	2.8E-2
	KEGG_PATHWAY	Axon guidance	RT		31	1.6	3.2E-3	3.1E-2
	KEGG_PATHWAY	Non-small cell lung cancer	RT		16	0.8	3.8E-3	3.5E-2
	KEGG_PATHWAY	Focal adhesion	RT		33	1.7	4.2E-3	3.7E-2
	KEGG_PATHWAY	HIF-1 signaling pathway	RT		21	1.1	4.4E-3	3.8E-2
	KEGG_PATHWAY	Rap1 signaling pathway	RT		34	1.7	4.5E-3	3.8E-2
	KEGG_PATHWAY	Autophagy - animal	RT		25	1.3	5.3E-3	4.4E-2
	KEGG_PATHWAY	Apoptosis	RT		24	1.2	6.7E-3	5.5E-2
	KEGG_PATHWAY	Yersinia infection	RT		24	1.2	7.4E-3	5.7E-2
	KEGG_PATHWAY	Basal cell carcinoma	RT		14	0.7	7.4E-3	5.7E-2
	KEGG_PATHWAY	Regulation of actin cytoskeleton	RT		34	1.7	7.9E-3	6.0E-2
	KEGG_PATHWAY	Endometrial cancer	RT		13	0.7	9.7E-3	7.1E-2
	KEGG_PATHWAY	Inositol phosphate metabolism	RT		15	0.8	1.1E-2	7.8E-2
	KEGG_PATHWAY	Cell cycle	RT		22	1.1	1.1E-2	7.8E-2
	KEGG_PATHWAY	C-type lectin receptor signaling pathway	RT		19	1.0	1.2E-2	8.0E-2
	KEGG_PATHWAY	T cell receptor signaling pathway	RT		19	1.0	1.2E-2	8.0E-2
	KEGG_PATHWAY	Type II diabetes mellitus	RT		11	0.6	1.2E-2	8.0E-2
	KEGG_PATHWAY	Longevity regulating pathway	RT		17	0.9	1.2E-2	8.0E-2

Figure S5. KEGG pathway analysis using predicted gene targets of deregulated microRNAs in SCCC. The predicted gene targets of miR-383-5p, miR-424-5p and miR-508-3p, all deregulated in SCCC versus healthy control tissue, were downloaded from TargetScan (release 8.0; https://www.targetscan.org). The genes were subsequently used as input for the database for annotation, visualization and integrated discovery (DAVID- 2021 update; https://david.ncifcrf.gov) for a KEGG pathways analysis. Listed are the top 50 most significant pathways.
